# Synthesis of Dimpled Particles by Seeded Emulsion Polymerization and Their Application in Superhydrophobic Coatings

**DOI:** 10.3390/membranes12090876

**Published:** 2022-09-11

**Authors:** Likui Wang, Florian Ion Tiberiu Petrescu, Jing Liu, Hongping Li, Gang Shi

**Affiliations:** 1 The Key Laboratory of Synthetic and Biological Colloids, Ministry of Education, School of Chemical and Material Engineering, Jiangnan University, Wuxi 214122, China; 2 Department of Mechanisms and Robots Theory, Bucharest Polytechnic University, 060042 Bucharest, Romania

**Keywords:** dimpled particles, seeded emulsion polymerization, fluoroacrylate, superhydrophobic

## Abstract

Dimpled particles are synthesized through the seeded polymerization of fluoroacrylate and styrene on swelled polystyrene spheres. The morphologies of the particles can be controlled by the polymerization temperature, the amount of solvent swelling the seeds or the ratio of the fluoroacrylate monomer over styrene. Golf-ball-like particles with many small dimples on their surfaces are obtained at low polymerization temperatures or with a small amount of solvent. Particles with a large single dimple are formed at higher polymerization temperatures, with larger solvent amounts or a higher ratio of fluoroacrylate over styrene. The morphology formation mechanism of these dimpled particles is proposed and the application of these particles in the fabrication of superhydrophobic coatings is demonstrated.

## 1. Introduction

The synthesis of anisotropic colloidal particles has attracted great interest due to their wide applications in the field of self-assembly [1,2,3,4,5], drug delivery [6,7], Pickering emulsion [8,9], superhydrophobic materials [10,11,12] and energy storage [13]. Particle shape anisotropy plays a significant role in directed self-assembly and the formation of complex structures rather than the close-packed colloidal crystals formed by isotropic particles [14,15]. Because of surface tension, most of the initially synthesized polymer particles are spherical. Fortunately, through seeded polymerization, the shape of particles could be engineered by utilizing the interaction between different polymer or solvent contents in the seed particles [1,16,17,18,19]. For example, during seeded emulsion polymerization, the different crosslinking degrees of the polymers lead to the extrusion of the newly formed phases and formation of dumbbell-like particles [20,21,22]. The difference in the hydrophilicity of polymers could also be employed to initiate phase separation for the preparation of particles with various shapes [23,24,25,26]. Additionally, the addition of a solvent is a widely adopted approach to produce particles with surface buckling during dispersion polymerization. The evaporation of the solvents in different domains during polymerization contributes to the localized contraction or deformation of the particles, forming a large variety of nonspherical particles, including particles with dents or wrinkles on their surfaces [27], disk-like and polyhedral particles, almond shell-like particles, void-containing particles, and rugby ball-like and red blood corpuscle-like hollow particles [28]. 

On the other hand, although fluorination of surfaces is widely adopted for the fabrication of hydrophobic surface, fluorinated monomers have seldom been employed in the seeded emulsion polymerizations [29,30,31,32,33,34,35]. Few articles have reported the synthesis of anisotropic particles by directly incorporating fluorinated monomer in the seeded polymerization [36]. Since fluorinated polymers are amphiphobic, it is expected that the addition of fluorinated monomers would enhance the incompatibility between different compositions, facilitating the formation of nonspherical particles. Herein, we study the emulsion polymerization of 1H, 1H, 5H-octafluoropentyl methacrylate (OFMA) and styrene (St), with swelled polystyrene (PS) spheres as the seeds. It is found that the combination of the fluoroacrylate and the swelling solvent results in dimples on the particle surface. The influence of the polymerization temperature, amount of swelling solvent and the content of fluoroacrylate are extensively studied. The dimpled particles are found to be applicable in fabricating superhydrophobic surfaces. 

## 2. Materials and Methods

### 2.1. Chemicals and Materials

St, OFMA and potassium persulfate (KPS) were purchased from Sigma-Aldrich, St. Louis, MO, USA. Azobis (isobutyronitrile) (AIBN), PX and ethanol were obtained from Sinopharm Chemical Reagent Co., Ltd., Shanghai, China. All chemicals were used as received without further purification, except that AIBN was purified by recrystallization in ethanol.

### 2.2. Synthesis of PS/P(OFMA-S) Composite Particles

PS spheres of 755 ± 11 nm were synthesized by emulsifier-free emulsion polymerization [37,38,39] and were used as seeds to prepare PS/P(OFMA-S) composite particles in the following seeded polymerization process, with the detailed recipe shown in Table 1. PS latex and a certain amount of PX were added into a 100 mL three-necked round-bottom flask. The mixture was stirred for 18 h at 300 rpm to allow PX to swell the PS spheres. Subsequently, the above emulsion was heated to a certain temperature with nitrogen bubbling, and a solution of AIBN dissolved in a mixture of St and OFMA was added. Except for in the group of experiments for comparison of the influence of polymerization temperatures, the polymerization temperature is 80 °C. After 12 h of polymerization, the composite particles were washed with deionized water and ethanol three times by repeated centrifugation and redispersion, and were finally collected after drying in a 60 °C oven overnight.

### 2.3. Characterizations

The morphologies of particles were characterized on a scanning electron microscope (SEM, Hitachi, Tokyo, Japan, S-4800, 20 kV) and the feature sizes of the particles were obtained by measuring at least 200 particles. The SEM samples were sputtered with Au to improve the conductivity. The hydrophobic property of particulate coatings was analyzed with an OCA (Optical Contact Angle) meter (Dataphysics, OCA40, Filderstadt, Germany) via the sessile drop method using the Laplace–Young fitting algorithm. The volume of the water drop was 3 μL and the dispensing speed was 0.5 μL/s. The contact angle values were determined from the average of three measurements at different positions. TGA curves were obtained with a Mettler TGA 1100 SF, and FTIR spectra were collected with ABB FTLA 2000-104 (ABB, Zurich, Switzerland). 

## 3. Results and Discussions

**Figure 1:**
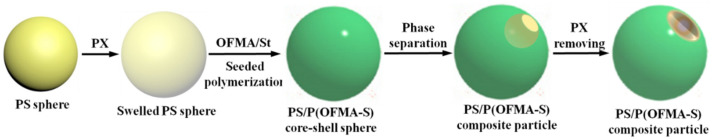
Synthesis of dimpled PS/P(OFMA-S) particles via seeded emulsion polymerization.

As illustrated in Figure 1, PS spheres are first swelled with para-xylene (PX) and subsequently used as seeds for emulsion polymerization of the mixture of OFMA and styrene. The fluorinated polymers produced by polymerization are amphiphobic, incompatible with the swelling solvent, leading to the phase separation of the PX with the polymers. Consequently, PX is squeezed out from the particles and dimples are formed on the sphere surfaces after removal of the solvent.

**Figure 2:**
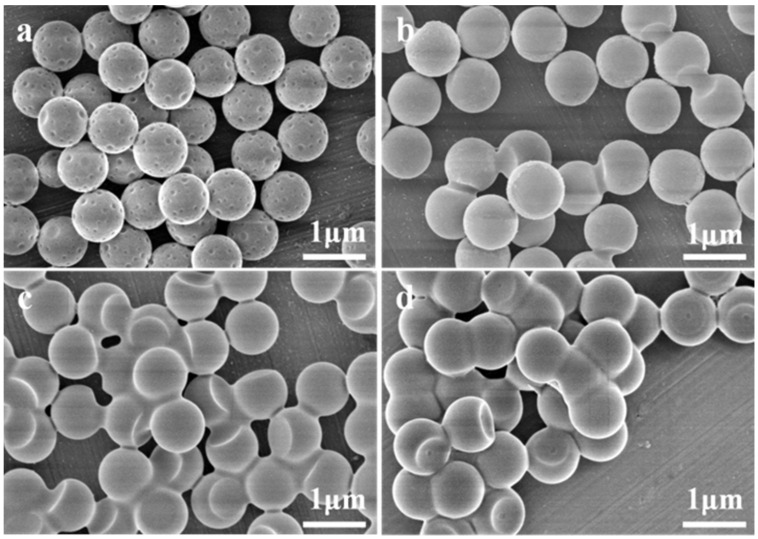
SEM images of PS/P(OFMA-S) composite particles synthesized with *w*_OFMA_/*w*_St_ of 1 and *w*_PX_/*w*_PS_ of 2 at different polymerization temperatures: (**a**) 60 °C, (**b**) 70 °C, (**c**) 80 °C, (**d**) 90 °C.

First, the influence of the polymerization temperatures on the morphologies of the particles was investigated. Figure 2 shows the PS/P(OFMA-S) composite particles synthesized from 775 nm PS spheres with *w*_OFMA_/*w*_St_ of 1 and *w*_PX_/*w*_PS_ of 2 at different temperatures. For the sample obtained at 60 °C, there are many small spherical indentations with average diameters of 150 nm around the particle surface. With the polymerization temperature elevated to 70 °C or above, a large indentation appears on the sphere surface instead of small indentations. The diameters of the single indentations for the samples synthesized at 70 °C, 80 °C are 455 nm and 625 nm, respectively. The ratios of the diameter of the indentation over that of the particles are 0.52 and 0.72, respectively. Before polymerization, PX is swelled into the PS spheres. During polymerization of OFMA and styrene with these swelled spheres as seeds, the incorporation of a fluorinated carbon chain transforms the polymer from oleophilic to oleophobic. PX tends to phase-separate from the polymer network and form droplets imbedded near the surface of the spheres, which decreases the Gibbs free energy of the systems. After completely drying, indentations are formed on the particles. It is speculated that at a low temperature, the swelled particles are resistant to shape deformation and the phase separation of the droplets could only happen locally. The localized solvent droplets on the sphere surface lead to the formation of small individual indentations. With the increase in the temperature, the spheres’ deformability become larger and the solvent inside the sphere tends to separate from the polymer and form a single droplet. Thus, the interface area between the polymer and the solvent is minimized, and so does the Gibbs free energy. Therefore, above 70 °C, most of the particles have a morphology with one big dent on their surface instead of multiple small indentations. However, at 90 °C, the relative size of the indentation decreased to 0.68, probably because the evaporation of the solvent is enhanced by the high temperature and a portion of PX escapes from the emulsion system before and during the polymerization of the monomer. The decrease in the solvent amount in the spheres causes the decrease in the relative size of the dents.

**Figure 3:**
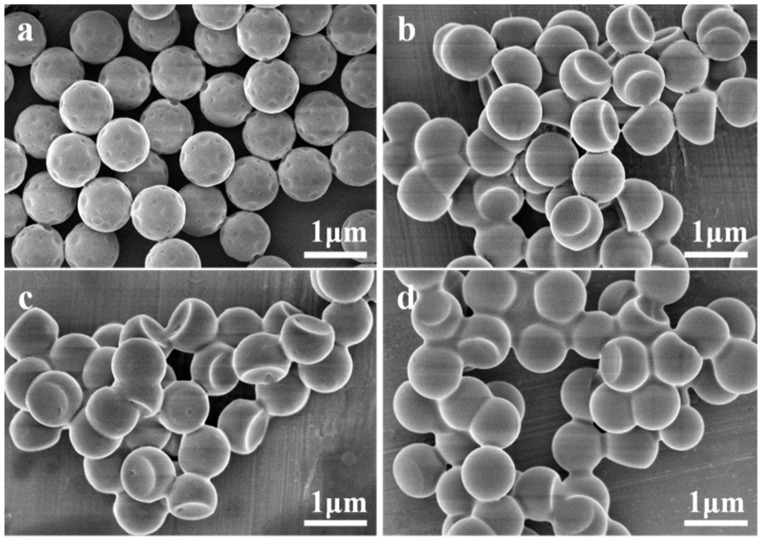
SEM images of PS/P(OFMA-S) composite particles obtained with constant *w*_OFMA_/*w*_St_ of 1 and different *w*_PX_/*w*_PS_: (**a**) 1, (**b**) 3, (**c**) 4, (**d**) 6 at 80 °C.

Figure 3 illustrates the SEM images of the composite particles synthesized from 775 nm spheres with different *w*_PX_/*w*_PS_ with a constant *w*_OFMA_/*w*_St_ of 1. With a *w*_PX_/*w*_PS_ of 1, small pits appear on every composite sphere, conferring the particles golf-ball-like morphology. With the increase in *w*_PX_/*w*_PS_ to 2, a large indentation could be observed on spheres instead of small pits. The ratios of the diameters of the indentation over that of the composite particles are 0.12, 0.72, 0.75, 0.796 and 0.801 for *w*_PX_/*w*_PS_ of 1, 2, 3, 4, 6, respectively. It could be observed that the solvent plays an important role on the morphology of the particles. The swelling of the particles by PX enhances the polymer segmental mobility and leads to a depressed *T*_g_. However, when *w*_PX_/*w*_PS_ is 1, the polymer segmental mobility might not be high enough for larger shape deformation. Consequently, PX is expelled from the polymer network to form small droplets near the surfaces, causing the formation of a large number of small pits. When *w*_PX_/*w*_PS_ is 2 or larger, the polymer chain segmental mobility is improved, allowing the formation of larger indentation. Additionally, the formation of single indentation is preferred to multiple small pits because the interface between the PX droplet and the fluorinated polymers is minimized. 

**Figure 4:**
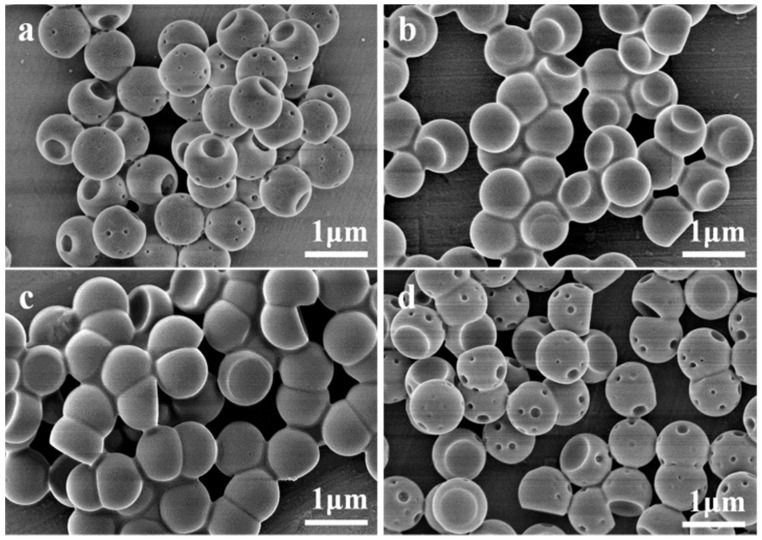
SEM images of PS/P(OFMA-S) composite particles prepared with a fixed *w*_PX_/*w*_PS_ of 4 and different monomer ratios *w*_OFMA_/*w*_St_ of (**a**) 1/4, (**b**) 1/2, (**c**) 2/1, (**d**) 4/1.

Figure 4 demonstrates the SEM images of PS/P(OFMA-S) composite particles with increasing ratios of *w*_OFMA_/*w*_St_. The ratios of the diameter of the indentation over that of the composite spheres are 0.44, 0.67, 0.796, 0.82 and 0.68 for *w*_OFMA_/*w*_St_ of 1/4, 1/2, 1/1, 2/1 and 4/1, respectively. With *w*_OFMA_/*w*_St_ of 1/4, the composite particles have one large dent as well as tiny indentations. Although all these syntheses are conducted with the same *w*_PX_/*w*_PS_, with the increase in *w*_OFMA_/*w*_St_, the solubility of PX in the particles decreases, meaning that more PX could be squeezed out from the particle and larger droplets could form on the surface of the spheres. Consequently, larger indentations are formed on the surface of the particles. However, with a further increase in the fluorinated polymer content to 4/1, small localized dents reappear in addition to the large one, which might be attributed to the enlargement of the glass transition temperature *T*_g_ induced by increased fluorine content [40], diminishing the deformability of the polymer spheres. This contradicts the trend of formation of a big droplet that requires larger deformation of spheres than formation of smaller localized dents on the surface.

**Figure 5:**
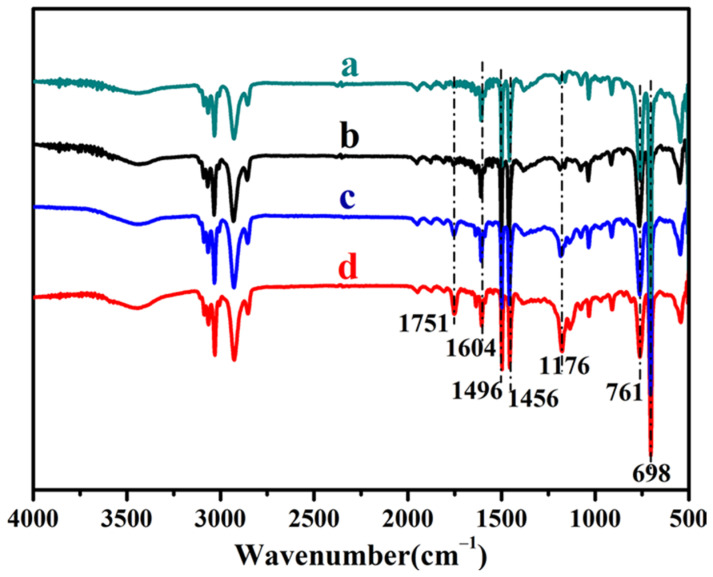
FTIR spectra of (a) PS spheres and the composite particles with different monomer ratios: *w*_OFMA_/*w*_St_ = (b) 1/4, (c) 1/1, (d) 4/1.

Figure 5 shows the FTIR spectra of the PS spheres and the composite particles prepared with different content of OFMA. The absorbance peaks at 1604 cm^−1^, 1496 cm^−1^, and 1456 cm^−1^ could be attributed to the vibration of benzene rings. The double peaks at 761 cm^−1^ and 698 cm^−1^ are the characteristic absorbance of monosubstituted benzene rings. The peaks at 3058 cm^−1^ and 3024 cm^−1^ arise from the stretching vibration of saturated C-H. For curves b to d, in the spectra of the composite particles, besides the absorption bands of the polystyrene segments, some new peaks appear. The absorption band at 1751 cm^−1^ belongs to the stretching vibration of carbon–oxygen double bond in ester groups. The vibration band of C-O-C bond in the ester group overlaps with the stretching band of the C-F bond at 1176 cm^−1^~1134 cm^−1^. With the increased amount of OFMA, the peaks at 1751 cm^−1^ and 1176 cm^−1^~1134 cm^−1^ intensify, which suggests the successful formation of copolymer of OFMA and styrene. 

**Figure 6:**
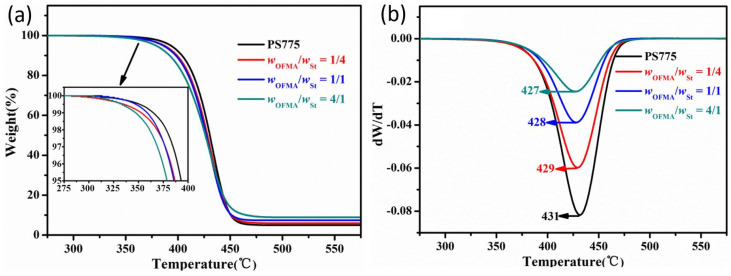
TGA (**a**) and DTG (**b**) curves of PS spheres and PS/P(OFMA-S) particles.

Figure 6 illustrates the TGA and DTG curves of the PS spheres and the composite particles synthesized with different contents of OFMA. With addition of OFMA, the temperature at 10% weight loss decreases from 404 °C to 399 °C, 398 °C and 392 °C with the *w*_OFMA_/*w*_St_ of 1/4, 1/1, and 4/1, respectively, and the temperature at 50% weight loss also decrease from 430 °C to 428 °C, 427 °C and 426 °C, respectively. It suggests that the introduction of OFMA enhances the decomposition of the polymer at the initial stages, mainly due to the lower thermal stability of acrylic groups in PS/P(OFMA-S) composite particles than that of the benzene groups in PS spheres. On the other hand, the residue left at 600 °C increases from 4.96% for PS spheres to 5.93%, 7.4% and 8.88% the *w*_OFMA_/*w*_St_ of 1/4, 1/1, and 4/1, respectively. Additionally, from the DTG curves we can observe that although the temperature of maximum weight loss decreases with the increase in OFMA content, the maximum weight loss speed decreases gradually. These should be attributed to the excellent thermal stability of fluoroalkyl groups inside the composite particles.

**Figure 7:**
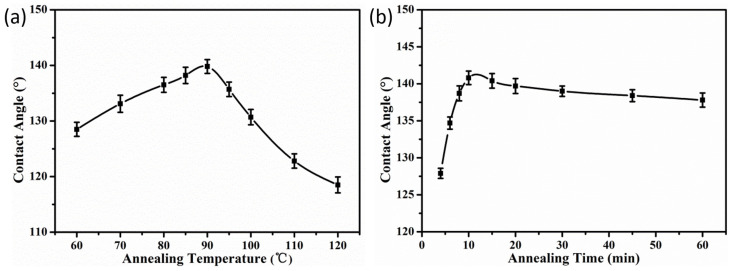
Effects of heat treatment on hydrophobic properties of coatings fabricated with PS/P(OFMA-S) composite particles: (**a**) at different annealing temperatures for 15 min, (**b**) with different annealing time at 90 °C.

The PS/P(OFMA-S) composite particles were spread on glass slides and annealed at different temperatures for 15 min. From Figure 7a, it could be observed that with the increase in the annealing temperature, the contact angle of the coatings gradually increases from 128.5° of 60 °C to 140.2° of 90 °C. This could be due to the higher segmental mobility of the fluorinated chains at higher temperatures, which tends to migrate from the bulk to the surface of the particles to minimize Gibbs free energy because of its oleophobic nature [41]. However, with the further increase in the temperature, the contact angles of the films decrease, probably owing to the fact that the coalescence of particles at temperatures near the glass transition temperatures reduces the surface roughness, which together with the surface nature determines the contact angles of a surface. As shown in Figure S1, it is noticed that after annealing at 120 °C, the coating becomes translucent, indicating the coalescence of the particles and partial loss of the porous structures. If the annealing temperature is fixed at 90 °C, the contact angle is enhanced to 140.9° with the elongation of the annealing time to 10 min, as shown in Figure 7b. However, if annealing time is further increased, the contact angle gradually decreases. This could also be attributed to the combinative effect of migration of fluorinated chains and reduction in surface roughness [34,42].

Moreover, the influence of OFMA content on contact angles was also investigated. As shown in Figure S2, with *w*_OFMA_/*w*_St_ of 1/4, 1/2, 1/1, 2/1 and 4/1, the contact angles of the annealed coating are 126.5°, 133.8°, 140.2°, 138°, 136.6°, respectively. With the increase in OFMA usage, the higher surface roughness induced by the enlarged dents on the particle surface and the elevated fluorinated chains content leads to the enlargement of the contact angle. With a further increase in OFMA content, although the fluorinated chain content is enlarged, the dent diameter decreases and leads to slight diminishment of surface roughness, causing the lowering of the contact angle. 

## 4. Conclusions

With PS spheres swelled with solvents as seeds, the mixture of OFMA and styrene was emulsion-polymerized, forming fluorinated composite particles with dents on their surfaces. The influence of the polymerization temperature, the amount of solvent and the ratios of OFMA over styrene in the monomer mixture on the morphologies of the composite particles were studied. At a lower polymerization temperature or with lower solvent usage, the PS/P(OFMA-S) composite particles were full of small dimples on their surface, conferring them golf-ball-like morphologies. At higher polymerization temperatures with larger solvent usage or higher OFMA/styrene ratio, a large dent formed on the particle surface instead of multiple dimples. The composite particles were employed to fabricate superhydrophobic coatings, and the influence of the annealing temperature and time were studied. It was found that with the particles prepared with *w*_OFMA_/*w*_St_ of 1/1, the contact angle of the coating could be 140.9°. These composite particles may be applied in the directional self-assembly and fabrication of hydrophobic surfaces.

## Figures and Tables

**Table 1:** Recipes for the preparation of PS/P(OFMA-S) composite particles.

PS Latex (2.5 wt%)/g	OFMA/g	St/g	AIBN/g	PX/g
40	0.2	0.8	0.01	2
40	0.333	0.667	0.01	2
40	0.5	0.5	0.01	2
40	0.667	0.333	0.01	2
40	0.8	0.2	0.01	2
40	0.5	0.5	0.01	1
40	0.5	0.5	0.01	3
40	0.5	0.5	0.01	4
40	0.5	0.5	0.01	6

## Supplementary Materials

The following supporting information can be downloaded at: https://www.mdpi.com/article/10.3390/membranes12090876/s1, Figure S1: Digital photos of glass slides coated with PS/P(OFMA-S) composite particles annealed at different annealing temperatures for 15 min and their static hydrophobic performances: (a,d) 60 °C; (b,e) 90 °C; (c,f) 120 °C.; Figure S2: Water contact angles of coatings with PS/P(OFMA-S) composite particles obtained with different *w*_OFMA_/*w*_St_ of 1/4, 1/2, 1/1, 2/1 and 4/1.
