# Supplementary material for: Synthesis of Dimpled Particles by Seeded Emulsion Polymerization and Their Application in Superhydrophobic Coatings
Source: Membranes (Basel). 2022 Sep 11;12(9):876. doi: 10.3390/membranes12090876 (PMC9504608; doi:10.3390/membranes12090876)
Supplement: Supplementary file 1 [file membranes-12-00876-s001.zip › membranes-1887762-supplementary.pdf]

membranes

Supplementary material for

Synthesis of Dimpled Particles by Seeded Emulsion Polymerization and Their Application in Superhydrophobic Coatings

Likui Wang [1,†], Florian Ion Tiberiu Petrescu [2,†], Jing Liu [1], Hongping Li [1] and Gang Shi [1,*]

[1] The Key Laboratory of Synthetic and Biological Colloids, Ministry of Education, School of Chemical and Material Engineering, Jiangnan University, Wuxi 214122, China
[2] Department of Mechanisms and Robots Theory, Bucharest Polytechnic University, 060042 Bucharest, Romania
* Correspondence: gangshi@jiangnan.edu.cn
† These authors contributed equally to this work.

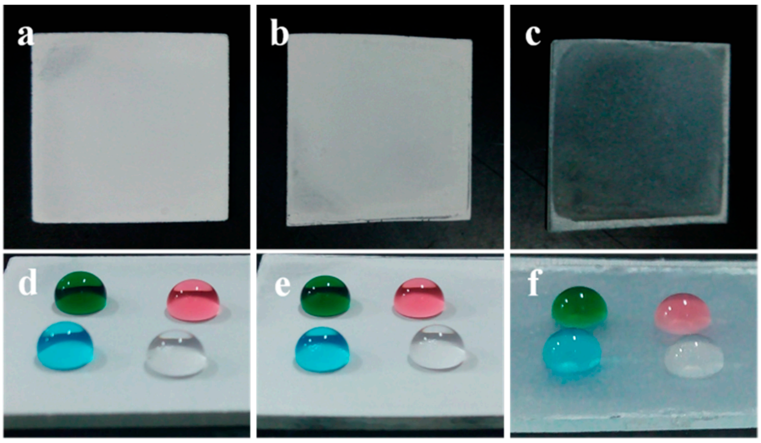

Figure S1. Digital photos of glass slides coated with PS/P(OFMA-S) composite particles annealed at different annealing temperatures for 15 min and their static hydrophobic performances: (**a**,**d**) 60 °C; (**b**,**e**) 90 °C; (**c**,**f**) 120 °C.

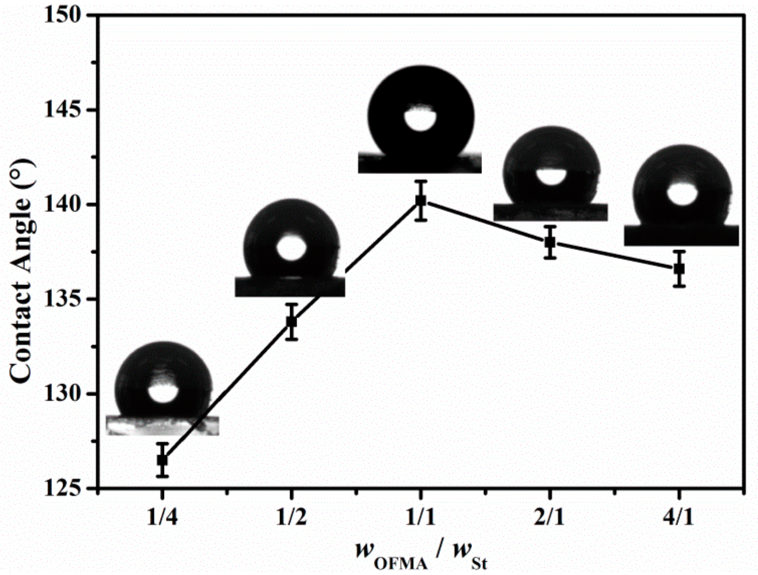

Figure S2. Water contact angles of coatings with PS/P(OFMA-S) composite particles obtained with different w_{OFMA}/w_{St} of 1/4, 1/2, 1/1, 2/1 and 4/1.
